# Three-dimensional characterization of adenomyosis in female mice: Toward a novel system for drug efficacy evaluation

**DOI:** 10.1210/jendso/bvag056

**Published:** 2026-03-17

**Authors:** Chihiro Ishizawa, Takehiro Hiraoka, Shizu Aikawa, Xueting He, Daiki Hiratsuka, Yamato Fukui, Mitsunori Matsuo, Tomoko Makabe, Gentaro Izumi, Miyuki Harada, Osamu Wada-Hiraike, Yasushi Hirota

**Affiliations:** Department of Obstetrics and Gynecology, Graduate School of Medicine, The University of Tokyo, Tokyo 113-8655, Japan; Department of Obstetrics and Gynecology, Graduate School of Medicine, The University of Tokyo, Tokyo 113-8655, Japan; Department of Obstetrics and Gynecology, Graduate School of Medicine, The University of Tokyo, Tokyo 113-8655, Japan; Department of Obstetrics and Gynecology, Graduate School of Medicine, The University of Tokyo, Tokyo 113-8655, Japan; Department of Obstetrics and Gynecology, Graduate School of Medicine, The University of Tokyo, Tokyo 113-8655, Japan; Department of Obstetrics and Gynecology, Graduate School of Medicine, The University of Tokyo, Tokyo 113-8655, Japan; Department of Obstetrics and Gynecology, Graduate School of Medicine, The University of Tokyo, Tokyo 113-8655, Japan; Department of Obstetrics and Gynecology, Graduate School of Medicine, The University of Tokyo, Tokyo 113-8655, Japan; Department of Obstetrics and Gynecology, Graduate School of Medicine, The University of Tokyo, Tokyo 113-8655, Japan; Department of Obstetrics and Gynecology, Graduate School of Medicine, The University of Tokyo, Tokyo 113-8655, Japan; Department of Obstetrics and Gynecology, Graduate School of Medicine, The University of Tokyo, Tokyo 113-8655, Japan; Department of Obstetrics and Gynecology, Graduate School of Medicine, The University of Tokyo, Tokyo 113-8655, Japan

**Keywords:** adenomyosis, mouse model, three-dimensional imaging, progestin

## Abstract

Adenomyosis is an intractable gynecological disorder that can give rise to severe pelvic pain, heavy menstrual bleeding, and infertility, predominantly affecting women of reproductive age. The limited efficacy of current pharmacological interventions in certain cases—particularly those resistant to hormone therapies—underscores the urgent need for deeper mechanistic insights and the development of novel therapeutic strategies. Progress in this field, however, has been hampered by the scarcity of suitable experimental models, as adenomyosis is primarily a human disease. In this study, we demonstrate a preclinical platform for therapeutic evaluation using a mouse model of mechanically induced adenomyosis, which recapitulates the key pathological features observed in humans. By integrating high-resolution three-dimensional (3D) tissue imaging, we successfully visualized the invagination and expansion of adenomyosis lesions spatiotemporally. This approach enabled the clear-cut and efficient assessment of progestin efficacy in situ. We propose that the combination of a mechanistically relevant mouse model and advanced 3D histological analysis constitutes a concise and robust system for therapeutic validation and compound screening, thereby facilitating the discovery of effective treatments for adenomyosis.

Adenomyosis is a benign gynecological disorder characterized by the presence of endometrial-like glandular epithelium and stromal tissue within the myometrium. The disease causes severe pelvic pain, heavy menstrual bleeding, and infertility in reproductive-age women [1-3]. Adenomyosis is generally considered an estrogen-dependent disease, as its pathogenesis is largely driven by estrogen upregulation [4, 5].

In the treatment of adenomyosis, progestins—synthetic hormones that mimic the effects of progesterone and counteract estrogen—are widely used [3, 6-9]. However, some cases exhibit progesterone resistance [5, 10-13]. While gonadotropin-releasing hormone (GnRH) agonists and antagonists are also effective in treating adenomyosis by suppressing estradiol secretion [4, 14, 15], long-term estrogen suppression has adverse health effects, limiting the duration of the treatments. Considering these limitations, there is an urgent need to develop an alternative therapeutic choice independent of hormonal treatment by elucidating the fundamental mechanisms of adenomyosis.

To date, various animal models have been developed to address the pathophysiology of adenomyosis. For example, neonatal tamoxifen exposure in mice has been used to develop adenomyotic lesions by inducing overwhelmingly estrogenic conditions [11, 13, 16-18]. For another, pituitary transplantation to the uterus has been shown to trigger the genesis of adenomyosis by the elevation of the local prolactin level [19, 20]. However, these models have low physiological relevance and little association with epidemiology. Several hypotheses have been proposed regarding the origin of adenomyosis, including the Müllerian rest theory, metaplasia theory, genetic mutation theory, and the endometrial invagination theory [21, 22]. Among them, the endometrial invagination theory that tissue injury and repair (TIAR) lead to the invagination of the endometrial tissue beyond the endometrial basalis into the myometrium [23] is widely accepted, as evidenced by the epidemiology [22, 24, 25]. Recently, a new hypothesis called endometrial–myometrial interface disruption (EMID) has been proposed to explain adenomyosis resulting from iatrogenic trauma to the endometrial–myometrial interface (EMI), describing the essential mechanisms underlying its pathogenesis [26, 27].

To mimic this, we have developed a mouse model of mechanically induced adenomyosis [28], providing a clinical relevance in terms of the uterine trauma following surgeries such as Caesarean section. Using the model, a previous study demonstrated that continuous activation of signal transducer and activator of transcription 3 (STAT3) is essential for the development of adenomyosis, with decreased adenomyosis formation in uterine-specific STAT3 knockout mice (*Stat3* uKO mice) [28]. Therefore, utilizing the model may provide a robust platform to evaluate drug efficacies.

In the earlier model, without exogenous estrogen supplementation after ovariectomy [28], lesion size and number remained relatively limited, allowing for quantification of lesions with conventional two-dimensional (2D) histology. However, under more physiological conditions with estrogen exposure, lesion expansion is expected to be greater, potentially complicating accurate evaluation by 2D sections alone. Importantly, advanced adenomyosis in humans has been reported to exhibit treatment resistance [3, 29-32], highlighting the clinical relevance of developing a system that can readily quantify extensive lesions. This limitation underscores the need for a three-dimensional (3D) approach that can capture the overall architecture of adenomyotic lesions.

Recently, studies using 3D imaging of the uterus have advanced the elucidation of embryo implantation in mouse models by immunostaining the uterine epithelium with E-cadherin [33-36]. Similarly, by adding α-smooth muscle actin (αSMA) staining in addition to E-cadherin, applying 3D imaging techniques to the adenomyosis study may facilitate understanding the pathogenesis and progression of adenomyosis with deeper spatial insights and enable an optical evaluation of therapeutic effects.

In this study, we performed 3D imaging of uteri from a mouse model of mechanically induced adenomyosis, demonstrating its usefulness in visualizing the process of lesion formation and evaluating the effectiveness of therapeutic drugs. To establish 3D imaging of adenomyosis, co-immunostaining of the epithelial marker E-cadherin and the myometrial marker αSMA was successfully achieved.

## Materials and methods

### Mice

This study used wild-type female mice (WT; C57BL/6N). All mice were housed in the University of Tokyo Animal Care Facility under specific-pathogen-free conditions with ad libitum feeding under a controlled environment (20-26 °C, 40-70% humidity, 12-h light/dark cycle, lights on at 8 Am). All experiments were approved by the animal experiment committee and the institutional review board of the committee of the University of Tokyo, in compliance with relevant guidelines and regulations (Approval nos.: A2023M165).

### Induction of mouse adenomyosis

All adult female mice from 7 to 16 weeks old underwent ovariectomy to eliminate the influence of the estrous cycle. More than 10 days after ovariectomy, the mice were injected with 100 ng/day/mouse of 17β-estradiol (NACALAI TESQUE, Japan) subcutaneously every 3.5 days. After two estradiol injections, mice were anesthetized with isoflurane and laparotomized with a median abdominal incision as previously reported [28]. One of the two uterine horns in each mouse was punctured 100 times per 1 cm using a 30 G needle (Dentronics, Japan) throughout the myometrium and endometrium, and the other uterine horn was evaluated as the control. Based on a previous report, 100 punctures were considered sufficient to injure the entire uterus throughout its full 3D extent [28, 37].

To landmark the operation range, both ends of the operation site were marked by 8-0 Vicryl (BEAR Medic, Japan). To prevent postsurgical adhesion, we placed an antiadhesive material (Seprafilm; Kaken Pharmaceutical Co., Ltd., Tokyo, Japan) around the injured site. After the operation, progesterone (4 mg/mouse/day), dienogest (5 mg/mouse/day), or oil was injected subcutaneously concomitantly with estradiol every 3.5 days. The mice were then sacrificed in specific postoperative periods, and both the injured and the non-injured uterine horns were collected for histological analysis. Estradiol dosage was decided according to the reports on the mouse sexual cycle repeats [7, 38]. Progesterone dosage (4 mg/mouse/day) and dienogest dosage (5 mg/mouse/day) were decided based on the reports [7, 39-41]. Dienogest was provided by Mochida Pharmaceutical Co., Ltd. All drugs were dissolved in 0.1 mL of sesame oil. The schedule of hormonal administration and operation was shown in the corresponding figures.

### Immunofluorescence

Formalin-fixed paraffin-embedded sections (6 μm) underwent immunofluorescence. After deparaffinization and hydration, the sections were subjected to antigen retrieval by autoclaving in 10 mmol/L sodium citrate buffer (pH 6.0) for 1 hour. After washing in PBS twice for 5 minutes each, the sections were blocked with blocking reagent (Dako Protein Block, Serum-Free, Ready-to-Use; X0909 1005094) for 1 hour. Next, sections were incubated overnight with primary antibodies including Antibodies to anti-E-cadherin (Cell Signaling Technology, Cat # 3199, RRID:AB_10691457, at a dilution of 1:200) and anti-α-Smooth Muscle Actin (Cell Signaling Technology Cat # 60839, RRID:AB_3720854, at a dilution of 1:50) in the dark. Nuclear staining was performed using 6-diamidino-2-phenylindole (DAPI) (Dojindo, at a dilution of 1:500). The images were captured by AXR (Nikon).

### 3D imaging

3D visualization of uterine samples was performed, following the techniques in the previous report [33]. To stain epithelial cells and smooth muscle, an anti-E-cadherin antibody (Cell Signaling Technology, Cat # 3199, RRID:AB_10691457, at a dilution of 1:200) and an anti-α-smooth muscle actin antibody (Cell Signaling Technology, Cat # 60839, RRID:AB_3720854 at a dilution of 1:50) were used. Samples were fixed in Dent's fixative (methanol: DMSO = 4:1) overnight at −20 °C and then washed with 100% methanol. The samples were bleached with 3% H_2_O_2_ in methanol at 4 °C overnight to remove pigmentation. After washing in PBST six times for 1 hour each, the samples were incubated with the indicated antibodies at room temperature on a rotator for 5 to 7 days in the dark. Following incubation, the samples were washed in PBST six times for 1 hour each at room temperature. They were then fixed in methanol for 30 minutes at room temperature. Finally, the tissues were cleared in BABB (benzyl alcohol: benzyl benzoate = 1:2) at room temperature for more than 1 hour in the dark. Z-stack confocal 3D images were acquired using AXR (version 5.30; Nikon) microscopes. Images were acquired using a 10× objective lens with a 3-µm Z-stack interval. To visualize the adenomyosis lesions in 3D, the Surface Tool in IMARIS (version 9.8, Oxford Instruments) was used. Surface reconstruction of the E-cadherin and αSMA regions was performed using automatic thresholding, enabling visualization and quantitative analysis of tissue morphology. The eutopic epithelium and adenomyosis epithelium were distinguished based on their spatial relationship to the myometrium. The epithelia within the myometrium were colored magenta, and eutopic epithelia were shown in green, by manually distinguishing the structures with reference to the orthogonal views.

### Measurement of volume of adenomyosis

The size of adenomyosis was measured by quantifying the volume of magenta-colored epithelial structures using IMARIS (version 10.2.0, Oxford Instruments).

### Statistics

All statistical analyses were performed using two-tailed Student's *t*-tests in GraphPad Prism 10. *P* values less than .05 were considered statistically significant.

## Results

### 3D visualization of mouse adenomyosis

**Figure 1:**
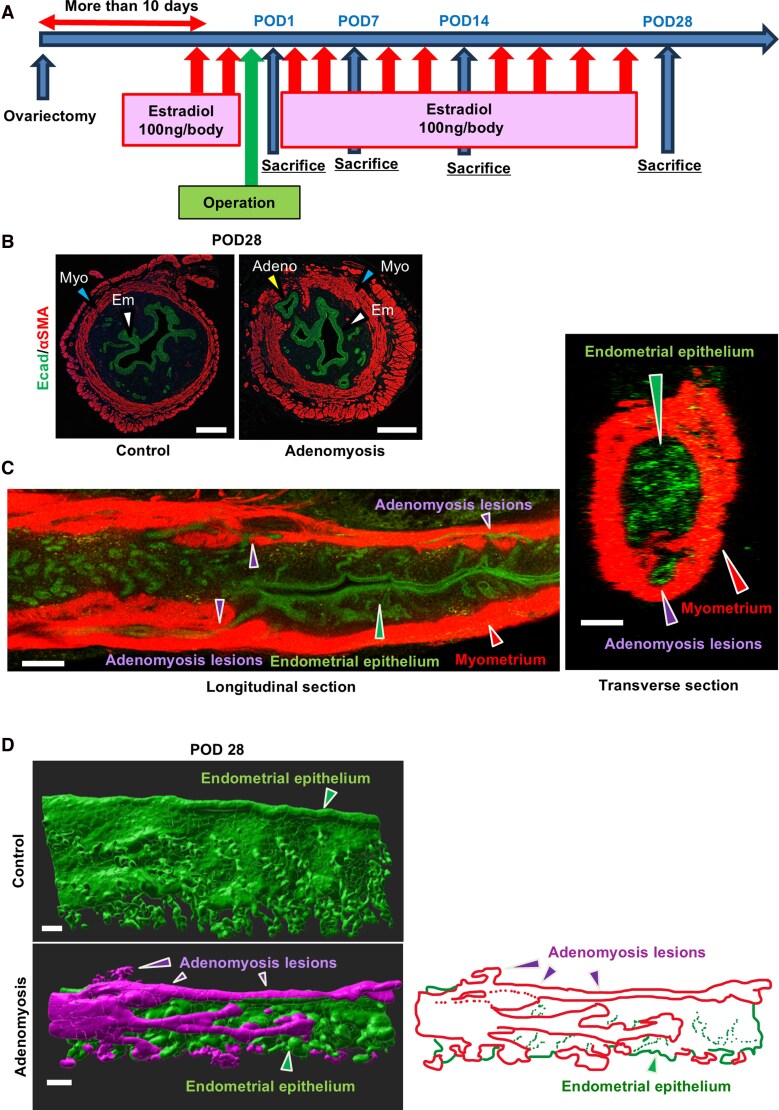
3D-visualization of adenomyosis. A The protocol of the study. B Representative results of the immunofluorescence for E-cadherin and αSMA on histological sections from control and adenomyosis groups. C Representative images of co-immunostaining for E-cadherin and αSMA, obtained from orthogonal views in the longitudinal and transverse planes. D Representative 3D-reconstructed images of whole mount immunofluorescence for E-cadherin. The eutopic endometrium was shown in green, and the adenomyosis lesions were depicted in magenta. Scale bar = 100 μm. Adeno, adenomyosis lesions; Em, endometrial epithelium; Myo, myometrium. More than four biologically different samples were examined in each analysis.

First, we performed ovariectomy to eliminate the hormonal effects of the estrous cycles. The uterine horns were then injured to induce adenomyosis lesions [28], and 17β-estradiol was administered to accelerate the development of lesions (Fig. 1A). On twenty-eight postoperative days after uterine injury (POD 28), adenomyosis lesions, the ectopic endometrial invasion in the myometrium, were observed by immunofluorescence of an epithelium marker E-cadherin and myometrial marker αSMA on histological sections (Fig. 1B), as previously reported [28].

Since 3D imaging is considered more informative than classical histological thin sections in capturing the thorough morphology of adenomyosis, we visualized adenomyosis lesions in 3D images by whole-mount immunofluorescence with clearing reagent and followed by 3D imaging by IMARIS. Anti-E-cadherin and anti-αSMA antibodies were used to label the uterine epithelium and myometrium, respectively.

Representative images of co-immunostaining for E-cadherin and αSMA, obtained from orthogonal views in the longitudinal and transverse planes, are shown in Fig. 1C. We identified adenomyosis lesions as ectopic endometrial invasion into the myometrium in the 3D-reconstructed images (Fig. 1D). The adenomyosis lesions were visualized in magenta in IMARIS, and the perspective views revealed that adenomyotic epithelium exhibited an intricate and continuous structure, consistent with the reports on human adenomyosis [42].

### Time-series 3D imaging reveals the developmental process of adenomyosis

**Figure 2:**
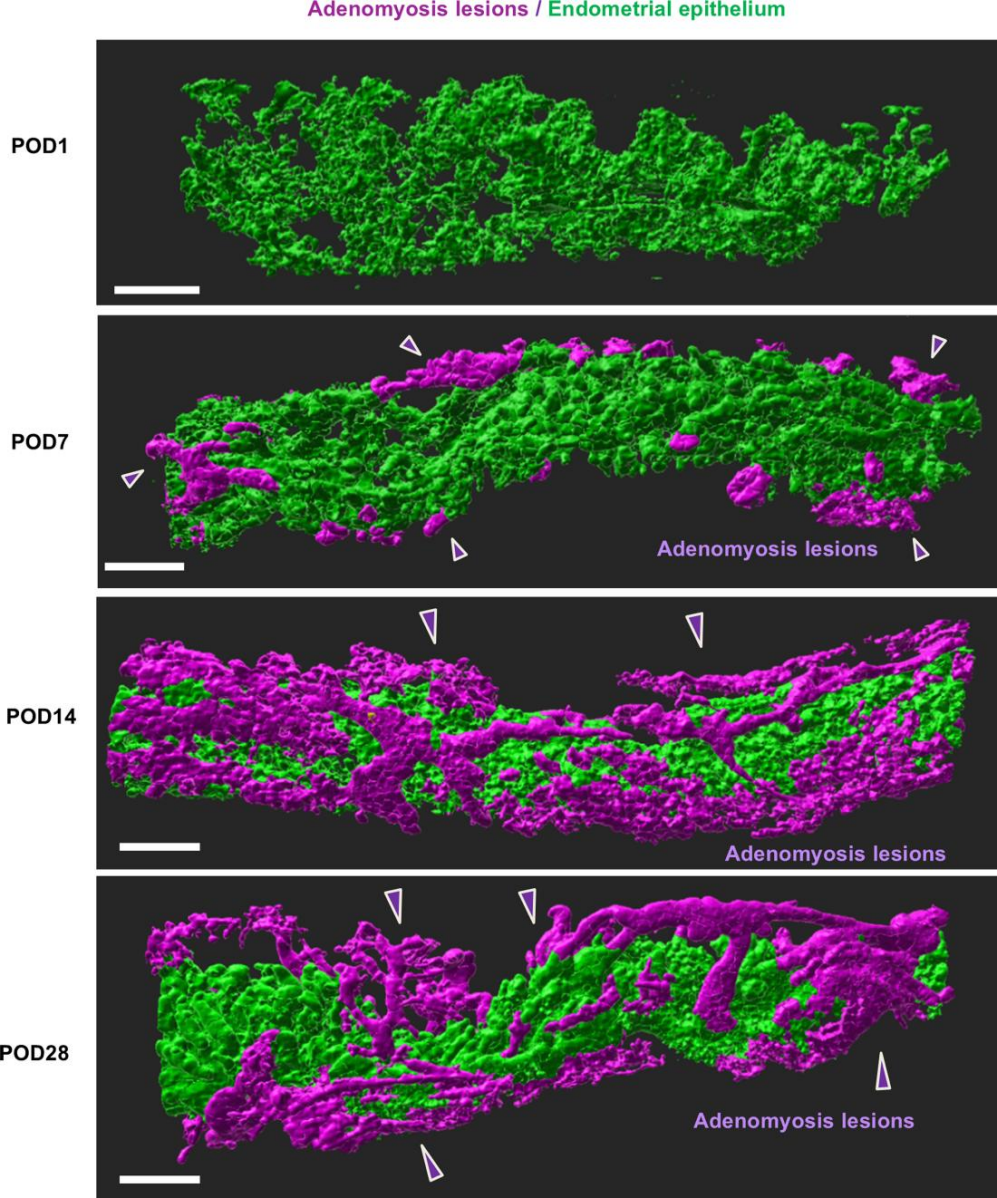
Time-series 3D imaging of the progression of adenomyosis. Time-series representative 3D-reconstructed images of whole mount immunofluorescence for E-cadherin. The eutopic endometria were shown in green, and the adenomyosis lesions were presented in magenta. Five biologically different uterine samples on POD 1, 7, 14, and 28 were investigated. Scale bar = 100 μm. For this figure, *n* = 5 per group.

Next, we sought to visualize the spatiotemporal developmental process of adenomyosis, which has never been demonstrated in prior studies using classical histological sections. We sacrificed mice on POD 1, 7, 14, and 28, and obtained 3D adenomyosis images at each time point (Fig. 2). As a result, the emergence of adenomyosis lesions was observed on POD 7, and the sporadic lesions extended within the myometrium in a continuous rhizome-like structure on POD 14. The completion of adenomyotic lesions was observed on POD 14 and POD 28 comparably (Fig. 2).

### 3D assessment of adenomyosis facilitates the evaluation of therapeutic efficacy

**Figure 3:**
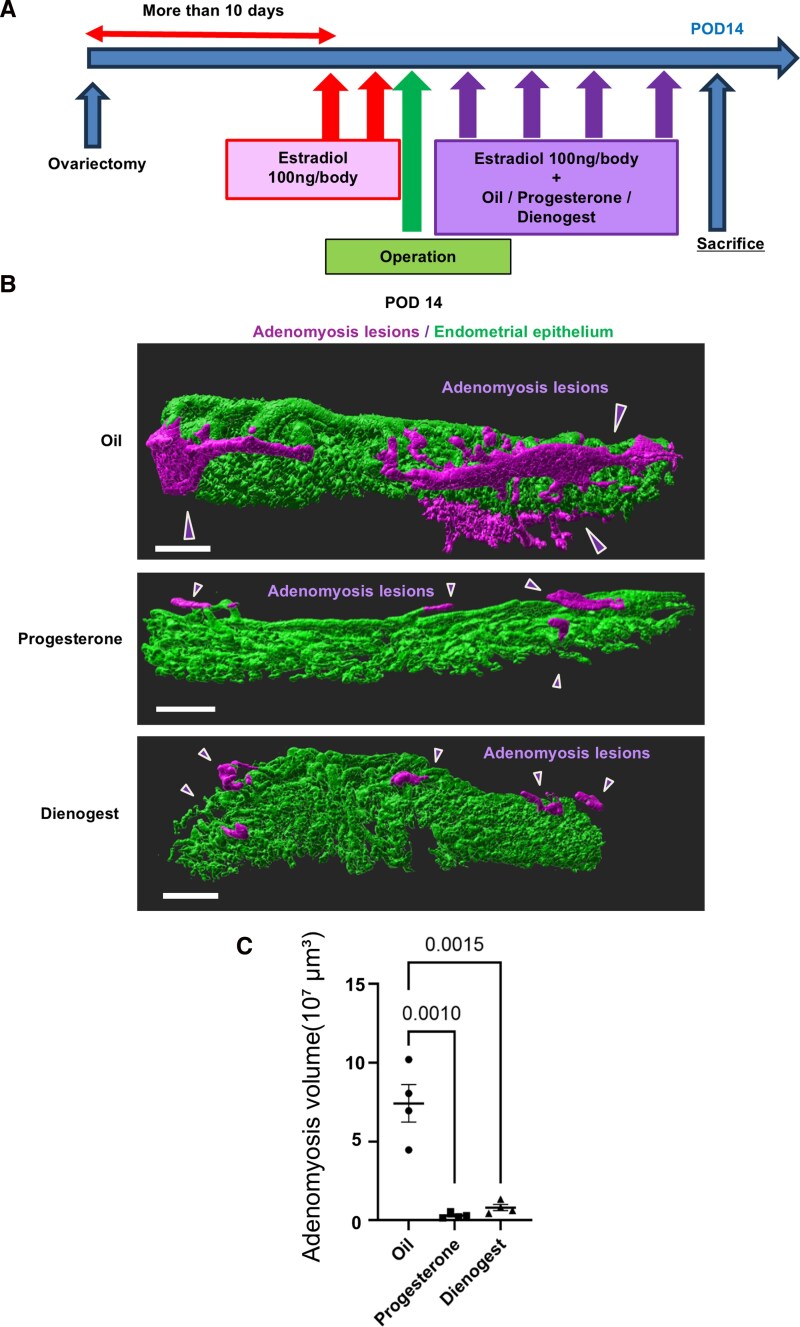
Evaluation of drug treatment of adenomyosis by 3D imaging. A The protocol of hormonal treatment. B Representative 3D-reconstructed images of whole-mount immunofluorescence for E-cadherin on POD 14. Decreased formation of adenomyosis lesions was visualized in the progesterone and dienogest groups than the oil group. Five biologically different uterine samples were used. Scale bar = 100 μm. C Quantification of adenomyosis volume showed a significant difference between the vehicle-treated adenomyosis group and the progesterone-treated group (*P* = .001), as well as between the vehicle-treated adenomyosis group and the dienogest-treated group (*P* = .0015). For this figure, *n* = 4 per group.

Because adenomyotic lesions are intricately generated within the myometrium, it has been challenging to accurately quantify the therapeutic efficacy of drugs for adenomyosis using conventional histological sections. Considering the visibility of 3D adenomyosis as demonstrated in the above results, we next set out to investigate the utility of the 3D evaluation of adenomyosis for evaluating the therapeutic effects of drugs. We administered progesterone (4 mg/day/mouse) and dienogest (5 mg/day/mouse) to mice in the adenomyosis group. Oil, progesterone, and dienogest were administered with estradiol after uterine injury in each group (Fig. 3A). Progesterone is widely known to be effective on adenomyosis [6-9], and dienogest, a progestin, an artificial progesterone product, is commonly used for the treatment of human adenomyosis. These time-series observations confirmed that the adenomyosis formation was completed on POD 14. Mice were then sacrificed on POD 14 and 3D imaging was performed to evaluate the therapeutic effects. As a result, while continuous rhizome-like structures were observed in the vehicle-treated adenomyosis, only sparse lesions were observed in either progesterone or dienogest-treated adenomyosis (Fig. 3B), with the difference distinguishably visualized by 3D imaging without a series of thin tissue slices. Next, we quantified the volume of adenomyosis, which showed a significant difference between the vehicle-treated adenomyosis group and the progesterone-treated group (7.42 ± 2.38 vs 0.30 ± 0.17 × 10^7^ μm^3^, *P* = .001), as well as between the vehicle-treated adenomyosis group and the dienogest-treated group (7.42 ± 2.38 vs 0.81 ± 0.39 × 10^7^ μm^3^, *P* = .0015) (Fig. 3C). These results suggest the utility of the 3D evaluation system of adenomyosis for the assessment of drug efficacy, proposing a concise drug screening system of adenomyosis.

## Discussion

In the present study, we established a 3D analytical platform for adenomyosis based on a mechanically induced mouse model [1]. This system provided superior morphological insight compared to conventional two-dimensional (2D) histological assessments reported in previous studies [28], allowing for clear visualization of the spatial architecture and temporal progression of adenomyotic lesions. Furthermore, we demonstrated its utility as a drug evaluation tool, eliminating the need for labor-intensive serial 2D sectioning. Although theoretically possible through reconstruction of immunostained 2D sections, the acquisition of a comparable volumetric dataset would require over 2000 individual 6-μm-thick slices, posing considerable technical and logistical challenges.

Several hypotheses have been proposed regarding the origin of adenomyosis, including the Müllerian rest theory, metaplasia theory, genetic mutation theory, and the endometrial invagination theory [21, 22]. The employed mouse model recapitulates subtype I adenomyosis, as defined by Kishi et al, wherein lesions originate from the endometrium and infiltrate the myometrium [25]. This model is also consistent with the EMID theory, which has been proposed to explain adenomyosis associated with disruption of the endometrial–myometrial interface [26, 27]. While it remains difficult to fully recapitulate adenomyosis arising from embryological processes or genetic alterations, or mutation-driven mechanisms, the present iatrogenic injury model reproduces key pathological features of adenomyosis development under an estrogen-dominant environment. Therefore, this model is considered to represent a trauma-associated mechanistic subtype of adenomyosis. Although adenomyosis and endometriosis share similarities in ectopic endometrial growth and pathogenic origins [10, 24], mechanical stimulation–driven pathogenesis according to the EMID theory appears to be more characteristic of adenomyosis.

While hormone-based therapies remain the standard clinical management, they are limited by side effects and suppression of ovulation, making them suboptimal, particularly in women desiring fertility preservation. Although the development of novel therapeutic agents, especially non-hormonal alternatives, is urgently sought, progress has been impeded by the absence of a reliable and reproducible system for therapeutic evaluation. Our 3D platform enables intuitive and immediate assessment of adenomyotic lesions, as demonstrated by its application in evaluating the efficacy of progesterone and dienogest. Progesterone is a physiological hormone produced endogenously in the body, whereas dienogest is a synthetic progestin used in clinical practice that exerts strong progestogenic activity with reported anti-estrogenic and anti-inflammatory effects. By employing both progesterone and dienogest, we evaluated physiological hormone responsiveness in vivo and assessed the therapeutic relevance of a clinically applied progestin. These findings support the utility of this model as a proof-of-concept platform for evaluating clinically relevant treatments for adenomyosis. This system thus holds promise as a preclinical screening tool for the identification of new drug candidates. In addition to screening drug efficacy, quantification of the disease enables the determination of the optimal drug dose through future dose-dependent efficacy experiments.

Beyond drug screening, the 3D imaging modality may facilitate molecular characterization of adenomyosis across its developmental timeline. Markers such as Ki67 and cluster of differentiation 31(CD31) have been shown to exhibit stage-dependent expression, distinguishing between lesion initiation and maintenance [37]. Additionally, key molecules implicated in adenomyosis pathogenesis—including KRAS, STAT3, progesterone receptor, estrogen receptor, cyclooxygenase-2 (COX-2), and nuclear factor-kappa B (NF-κB)—can be spatially mapped using whole-mount immunostaining [8, 10, 11, 43]. Several ligands in the STAT3 signaling pathway, such as Interleukin-6 (IL-6), Interleukin-6 (IL-10), oncostatin M (OSM), epidermal growth factor (EGF), vascular endothelial growth factor (VEGF), and heparin-binding EGF-like growth factor (HB-EGF), are also required for lesion development [44-48]. Visualizing these molecules at defined disease stages—initiation, progression, and maintenance—may reveal spatiotemporal molecular mechanisms underlying lesion formation and uncover stage-specific therapeutic targets.

In the previous adenomyosis model without exogenous estrogen supplementation after ovariectomy [28], lesion formation tended to be limited in size and number, allowing quantification even with conventional 2D histology. In contrast, our present model physiologically employed estradiol exposure, which promoted lesion expansion and resulted in the formation of continuous and interconnected lesions. Such extensive lesions are reminiscent of advanced-stage adenomyosis in humans, which has been associated with severe symptoms and poor reproductive outcomes [1, 3, 29-32, 49]. Importantly, these continuous lesions are difficult to evaluate accurately by 2D sections alone, underscoring the necessity of 3D observation for precise assessment of disease progression and therapeutic response. Given that widespread and advanced adenomyosis may contribute to progesterone resistance or reduced responsiveness to medical therapy [1, 3, 29-32], our 3D system could provide a unique platform to visualize and quantify disease severity and predict treatment outcomes.

Nonetheless, this study has several limitations. While the 3D reconstruction technique excels in depicting global tissue architecture, the resolution of its 2D slice image remains inferior to that of conventional thin-section histology. Detailed cellular morphology, for instance, is better evaluated through hematoxylin and eosin (H&E) staining of paraffin-embedded sections. Additionally, accurate localization of immune cell populations may be compromised by tissue autofluorescence inherent to whole-mount imaging. Antibody penetration in thick tissues also remains a challenge, particularly for proteins expressed in deep regions, and depends heavily on the efficiency of tissue clearing. Moreover, the applicability of our findings may be limited to subtype I adenomyosis, as the mouse model employed does not capture the full spectrum of other human adenomyosis subtypes.

In summary, we present a concise, reproducible, and robust 3D evaluation system for adenomyosis using a relevant murine model. This platform provides a comprehensive visualization and quantification of lesion morphology and progression, and holds potential for both therapeutic evaluation and mechanistic investigation. Its application to compound screening may accelerate the development of novel treatments for adenomyosis.

## Data Availability

All data generated or analyzed during this study are included in this published article.
